# Should alcohol-based handrub volume be customized according to the size of healthcare workers’ hands?

**DOI:** 10.1186/2047-2994-4-S1-P302

**Published:** 2015-06-16

**Authors:** F Bellissimo-Rodrigues, H Soule, A Gayet-Ageron, Y Martin, D Pittet

**Affiliations:** 1 Infection Control Programme, University of Geneva Hospitals, Switzerland; 2 Collaborating Centre on Patient Safety, World Health Organization, Geneva, Switzerland

## Introduction

Hand hygiene promotion is the most important tool to prevent health-care associated infections. While hand hygiene compliance has been well studied and promoted for the last 20 years, less attention has been devoted to study the quality of hand hygiene action.

## Objectives

We aimed to evaluate the amount of alcohol-based handrub (ABHR) needed to ensure appropriate hand antisepsis, depending on the healthcare workers (HCW) hand size.

## Methods

This was an experimental study based on the microbiological laboratory, evaluating 15 healthy HCWs with different hand sizes performing hand hygiene following the World Health Organization (WHO) recommended sequence, with 6 different volumes of 2-propanol 60% hand rub. According to the European Norm 1500 (EN1500) standard, bacterial count was measured on the HCWs’ finger tips at baseline, after one friction without ABHR, then after each application of ABHR from 0.5mL to 3mL. The primary outcome was the log_10_ reduction measured after each AHBR application. Generalized linear mixed models were performed to analyze the results.

## Results

Among the 15 participants, 10 were female (66.7%), 4 had small hands, with a mean (±SD) hand surface area (HSA) of 332.9 ± 22.2cm^2^, 6 had medium size hands (mean HSA= 404.2 ± 9.7cm^2^) and 5 had large hands (mean HSA= 473.2 ± 40.4cm^2^). The log_10_ reduction was significantly decreased for each supplemental 0.5ml of AHBR (0.28 log_10_; 95%CI: 0.23 to 0.34, p<0.001) after adjustment on hand size and baseline log_10_ count. The log_10_ reduction was significantly lower for large hands compared to small hands (-1.19 log_10_; 95%CI: -1.61 to -0.76, p<0.001), and significantly lower for medium hands compared to small hands (-0.57 log_10_; 95%CI: -0.98 to -0.15, p=0.007). HCWs with large hands achieved a mean reduction of only 1.42 log_10_ ± 1.31, after rubbing their hands with 3mL of ABHR.

## Conclusion

These results suggest the need of customizing the volume of ABHR used for hand hygiene, according to the size of the HCWs’ hands, for ensure appropriate hand antisepsis and patient safety.

## Disclosure of interest

None declared.

